# *In Vitro* Synergistic Activity of Combinations of Tetrahydroisoquinolines and Treatment Antibiotics against Multidrug-Resistant *Salmonella*

**DOI:** 10.1155/2023/6142810

**Published:** 2023-12-13

**Authors:** Rita Ayuk Ndip, Joelle Ngo Hanna, James Ajeck Mbah, Stephen Mbigha Ghogomu, Moses Njutain Ngemenya

**Affiliations:** ^1^Department of Biochemistry and Molecular Biology, University of Buea, P.O. Box 63, Buea, Cameroon; ^2^Department of Chemistry, Faculty of Science, University of Douala, P.O. Box 24157, Douala, Cameroon; ^3^Department of Chemistry, Faculty of Science, University of Buea, P.O. Box 63, Buea, Cameroon; ^4^Department of Medical Laboratory Sciences, University of Buea, P.O. Box 63, Buea, Cameroon

## Abstract

The global burden of *Salmonella* infections remains high due to the emergence of multidrug resistance to all recommended treatment antibiotics. Tetrahydroisoquinolines (THIQs) have demonstrated promising activity against multidrug-resistant (MDR) *Salmonella* Typhi. Hence, their interaction with treatment antibiotics was investigated for possible synergy. Twenty combinations of five THIQs (**1**, **2**, **3**, **4,** and **5**) and four antibiotics were tested against each of 7 *Salmonella* isolates by the checkerboard method giving a total of 140 assays performed. Fractional inhibitory concentration indices (FICIs) were calculated, and isobolograms were plotted. In terms of FICI, synergism ranged from 0.078 to 0.5 and the highest magnitude (0.078) was recorded for chloramphenicol-THIQ **1** combination. In a total of 140 antibiotics-THIQs combination assays, 27 were synergistic (17%), 42 were additive (30%), 11 were antagonistic (7.8%), and 60 were indifferent (42%). The synergistic activity recorded for each antibiotic class in combination based on the total of 7 bacterial isolates tested ranged from 14.29% to 71.43%; the highest percentage was recorded for two combinations (chloramphenicol or sulphamethoxazole with THIQ **1**). Ciprofloxacin-THIQ **1** combination showed additivity on all bacteria isolates tested (100%). Overall, THIQ **1** was the most synergistic and most additive in combination with three antibiotics (ampicillin, chloramphenicol, or sulphamethoxazole-trimethoprim). Some combinations of the THIQs and treatment antibiotics have shown high synergism which could potentially be efficacious against multidrug-resistant *S.* Typhi, hence this interaction should be further studied *in vivo*.

## 1. Introduction

The morbidity and mortality of salmonellosis in humans remain high [1]. It is caused by both typhoidal and nontyphoidal *Salmonella* serovars [2]. *Salmonella* is spread mainly by the consumption of contaminated food or water, poultry (chicken), and animal products such as eggs. It is transmitted through the faecal-oral route. The spread of *Salmonella* can be prevented through several strategies including vaccination and more effectively by improved water and food hygiene and proper cooking of food. Prevention of *Salmonella* infection from eggs and chickens to humans can be achieved by inclusion of antibiotics in animal feed and water and thorough washing of hands after handling and properly cooking these items before consumption [3].

Initially, treatment of *Salmonella* infections made use of penicillins, phenicols, cephalosporin, antifolates, and macrolides [4] and later following the emergence of resistance, fluoroquinolones and third generation cephalosporins were used as alternatives. However, high multidrug resistance to all the above mentioned treatment antibiotics has been reported worldwide, especially for *S.* Typhi [5]. Mild *Salmonella* infections are treated by electrolyte replacement and rehydration but severe cases require antibiotics [3]. Presently, the recommended treatment antibiotics are ciprofloxacin, ceftriaxone, and azithromycin, taking into consideration the local pattern of resistance [6]. However, genetic resistance to these antibiotics has been reported with decreased susceptibility and treatment failure due to MDR strains [7]. A recent study detected a high level of multidrug resistance to first-line antibiotics encoded by resistance marker genes (*tem*, *sul*1, and *dfr*A1) and also found low to moderate resistance to fluoroquinolones alongside single and double point mutations in the quinolone resistance determining region (QRDR) in *gyr*A [8]. The decreasing efficacy to the antibiotics in current use justifies the urgent search for new safe efficacious antibacterials and/or alternative strategies of their use against MDR *Salmonella* infection.

There are several reports of synergism in combination studies of natural or synthetic compounds with treatment drugs against various diseases including bacterial infections [9] and also studies on treatment drugs only such as antibiotic combinations [10]. So, combination therapy is presently one of many strategies used to counter antibiotic resistance. Several fixed dose combinations of antibiotics are in clinical use [11]. These combinations offer higher efficacy against multidrug-resistant bacterial strains due to their synergistic effects; they also have a broader spectrum of activity and reduce the risk of emergence of resistance during treatment [12]. Findings from antibacterial combination studies to counter MDR strains have been promising. Higher efficacy has been recorded in such studies compared to monotherapy where emergence of resistance to a single drug is more likely to occur [13]. Reports of combination of aminoglycosides with other antibiotics or plant-derived compounds have shown enhanced bactericidal activity against *Salmonella enterica* [14–16]. Also, synergistic activity has been recorded for some of the first-line anti-*Salmonella* antibiotics (ampicillin, chloramphenicol, and cefotaxime) in combination with an aminoglycoside (gentamycin) against MDR *Staphylococcus aureus* [17].

Tetrahydroisoquinolines (THIQs) are aromatic alkaloids which occur widely in nature. Their semisynthetic and synthetic derivatives have a broad range of bioactivity [18]. The tetrahydroisoquinoline contains a nitrogen heterocycle found in many approved drugs in clinical use for several diseases. Medicinal chemistry exploration of this scaffold has yielded analogues with antitubercular, antibacterial, antifungal, and antiviral action [19]. In a recent study of seventeen THIQs, six demonstrated a moderate but structure-related antibacterial activity against MDR *S.* Typhi strains comparable to some of the treatment antibiotics [20]. The findings on synergistic interactions mentioned above [9, 10] support the search for new efficacious antibacterial treatments against resistant strains using combination studies. Hence, this study investigated the interaction between these moderately active THIQs and some of the treatment antibiotics against MDR *S.* Typhi.

## 2. Materials and Methods

### 2.1. Test Microorganisms

Six MDR clinical isolates of *S.* Typhi (BU02, BU05, BU07, BU09, BU11, and BU70) were isolated from patient specimens obtained from health facilities in the South West Region, Cameroon. They had been characterized in an earlier study using cultural, biochemical, and molecular techniques, and the data on their susceptibility were published [8]. One control strain (*S.* Typhimurium ATCC 14028) obtained from the American Type Culture Collection, Manasses, USA, was included making a total of seven strains used in this study. Stocks of isolates were stored in 50% glycerol in Muller–Hinton broth (MHB) at −20°C.

### 2.2. Chemicals and Reagents

The materials, chemicals, and reagents used were Salmonella-Shigella agar, Mueller–Hinton agar, and broth from Liofilchem (Italy). Commercial antibiotics discs (ampicillin, chloramphenicol, sulphamethoxazole-trimethoprim, and ciprofloxacin) were purchased from Abtek Biologicals Ltd., UK. Dimethylsulfoxide (DMSO) was purchased from Sigma-Aldrich (USA). The tetrahydroisoquinolines were synthesized and obtained as solids in the Department of Chemistry, University of Buea, characterized as previously reported and stored at room temperature [20].

### 2.3. Instrumentation

To achieve the study objectives, the following equipment were used: incubator (DHP-9052, England), micropipettes (Accumax, India), and microplate reader (Emax microplate reader, Molecular Devices, USA). Microtitre plates (96 wells flat bottomed) were obtained from Thermo Fisher Scientific, Singapore.

### 2.4. Tetrahydroisoquinolines

**Figure 1:**
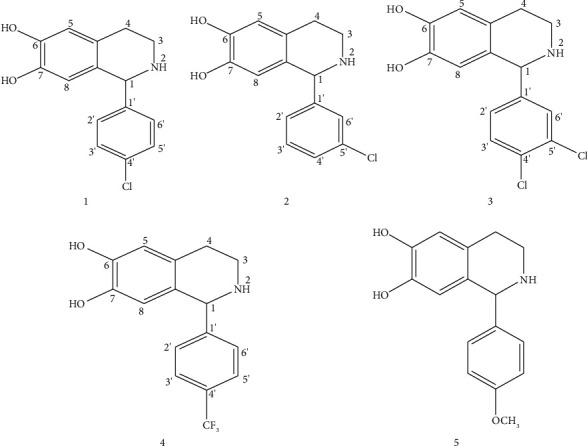
Chemical structures of 1-aryl-6,7-dihydroxy-1,2,3,4-tetrahydroisoquinolines. **1**: 1-(4-chlorophenyl)-6,7-dihydroxy-1,2,3,4-tetrahydroisoquinoline. **2**: 1-(3-chlorophenyl)-6,7-dihydroxy-1,2,3,4-tetrahydroisoquinoline. **3**: 1-(3,4-dichlorophenyl)- 6,7-dihydroxy-1,2,3,4-tetrahydroisoquinoline **4**: 1-(4-*α*,*α*,*α*-trifluoromethylphenyl)-6,7-dihydroxy-1,2,3,4-tetrahydroisoquinoline. **5**: 1-(4-*α*,*α*,*α*-trifluoromethoxyphenyl)-6,7-dihydroxy-1,2,3,4-tetrahydroisoquinoline.

Five tetrahydroisoquinolines (THIQs **1**, **2**, **3**, **4,** and **5**) used in this study were synthesized using the Pictet–Spengler reaction as described in detail [20]. In brief, a mixture of 3,4-dihydroxyphenethylamine hydrochloride (1 equiv), substituted benzaldehydes (1 equiv), and triethylamine (1 mL) in ethanol (10 mL) was stirred, heated under reflux (6–10 hours), and concentrated to remove the solvent. The residue was diluted with methylene chloride (100 mL) and distilled water (100 mL), giving a solid precipitate as the final product. The solid was collected by filtration, washed with acetone, and air-dried. The synthesis and structural characterization including all spectroscopic data of these five THIQs have been published in an earlier study of their antibacterial activity [20]. Their chemical structures are shown in Figure 1. Stock solutions of each compound were prepared by dissolving in dimethylsulfoxide (DMSO) as described in [20] and were further diluted in MHB before use in the experiments.

### 2.5. Determination of the Antibacterial Activity of Compounds

The diameters of zones of inhibition and the minimum inhibitory concentrations (MICs) of the antibiotics and selected THIQs had earlier been determined using disc diffusion and microdilution methods in a separate study, and the data were published in detail [20]. In brief, stock solutions (1.024 mg/mL) were prepared as described in the cited work and incubated at final concentrations from 1 to 512 *μ*g/mL with Salmonella bacterial cells (5 × 10^5^ CFUs/mL final density) in a 96-well microtitre plate in duplicates. Positive and negative controls were included and the optical densities (ODs) were read at 595 nm using a microplate reader (Emax microplate reader, Molecular Devices, USA). The plate was incubated at 37°C (DHP-9052, England) for 24 h, read visually for inhibition, and the OD read again at 595 nm. MIC was taken as the lowest concentration well with more than 50% inhibition of bacterial growth. MICs of selected antibiotics (ampicillin, chloramphenicol, sulphamethoxazole-trimethoprim, and ciprofloxacin) were determined as described above.

### 2.6. Determination of MIC and Interactions of THIQ-Antibiotic Combinations

The interactions of 5 THIQs with 4 antibiotics, i.e., 20 combinations each against 7 MDR *Salmonella* isolates giving a total of 140 assays, were assessed by the checkerboard method in 96-well microtitre plates as previously described [21]. Stock solutions, 8 times the MIC (8MIC) of antibiotics and THIQs, were prepared by dissolving in dimethylsulfoxide (DMSO) as described [20] and further diluted in MHB. Working solutions of each test substance were prepared by dilution of the stocks in MHB in separate microtitre plates. The antibiotic was serially diluted in 8 columns (2–9) (8MIC–MIC/16, 100 *μ*L per well, respectively), with concentration decreasing from wells A to H of each column. The THIQ was also diluted the same as the antibiotic in 8 rows (A–H) with concentration decreasing from wells 9 to 2 of each row. Then, the checkerboard assay was set up in a fresh plate by adding 50 *μ*L of diluted antibiotic to the corresponding well in a fresh plate, followed by 50 *μ*L of THIQ and 100 *μ*L of bacterial suspension giving final concentrations of 2MIC–MIC/64 and 5 × 10^5^ CFUs/mL for test substance and bacteria cells, respectively, in the 8 × 8 matrix of different concentrations. Antibiotic and THIQ were each included alone in columns 1 and 10, respectively, to determine their MICs.

The plates were incubated at 37°C (DHP-9052, England) for 24 h, and the optical density (OD) was measured at 595 nm (Emax microplate reader). The fractional inhibitory concentration (FIC) and FIC index (FICI) of each THIQ-antibiotic combination were calculated from the MICs of the THIQ or antibiotic alone and in combination according to the following equations:

(1) $$\begin{array}{c} \text{FIC}=\frac{\text{MIC of test compound in combination}}{\text{MIC of test compound alone}},\\ \text{FICI}=\text{FIC }\left(\text{antibiotic}\right)+\text{FIC}\left(\text{THIQ}\right). \end{array}$$

*In vitro* interactions were determined algebraically using the formula above and the nature of the THIQ-antibiotic interaction based on the combined and individual antimicrobial activities were interpreted based on the following cutoffs [22]: synergy: FICI ≤ 0.5, additive: 0.5 < FICI ≤ 1, indifference or no interaction: FICI: 1–4, and antagonism: FICI > 4.

### 2.7. Statistical Analysis

Five THIQs were combined with four antibiotics giving a total of twenty combination pairs. For each combination, the proportion of the total of 7 MDR *Salmonella* strains which showed a given type of interaction was calculated using the following formula:

(2) $$\begin{array}{c} \text{Percentage }\left(\%\right)\text{ of interaction}=\frac{x}{n}\times 100, \end{array}$$

where “*x*” is the number of each type of interaction against MDR *Salmonella* strains and “*n*” is the total number of MDR *Salmonella* strains tested (*n* = 7).

The overall percentage drug interaction type for all the 140 combination assays performed was calculated using the following formula:

(3) $$\begin{array}{c} \text{Overall percentage }\left(\%\right)\text{ interaction}=\frac{x}{y}\times 100, \end{array}$$

where *x* = number of drug interaction type and *y* = total number of drug combinations assays (140).

To determine the nature of their interaction, isobolograms were plotted using FICs of wells along the diagonal of the plate in which the concentrations of the two test compounds were decreasing and increasing, respectively, along the diagonal. FICs of antibiotic were plotted against the FICs of the THIQ and isobolograms which showed a concave curve indicate synergism; a convex curve indicates antagonism and linear curve shows additivity [23]. All statistical analyses were performed using GraphPad Prism version 8.40 (GraphPad Software, USA).

## 3. Results

### 3.1. Types and Effect of Interaction on the Minimum Inhibitory Concentration

The MICs of the antibiotics alone recorded in this study (16–256 *μ*g/mL) confirm that the *Salmonella* isolates were multidrug resistant. The antibacterial interactions were determined following the values of the FICI mentioned above [22]. Of an overall total of 140 combination assays, 27 were synergistic (17%) based on FICI ranging from 0.078 to 0.5, with the lowest and most synergistic being chloramphenicol with THIQ **1** (0.078) (Table 1).

The MICs of antibiotics in the combinations were considerably reduced compared to the MICs of the antibiotics alone. The reductions were much greater in the synergistic than the additive interactions. The highest reduction was observed for chloramphenicol with **1** and sulphamethoxazole-trimethoprim with **1**, which both caused a 64-fold reduction in the MIC of the antibiotics to 2 and 4 *μ*g/mL, respectively. Sulphamethoxazole-trimethoprim with **3**, ampicillin with **1**, and ciprofloxacin with **2** caused a 32-, 16-, and 16-fold decrease in the antibiotic MICs, respectively. The lowest reduction was ciprofloxacin with **4**. The reductions in the MICs for the additive and indifferent combinations ranged from 1- to 2-fold (Table 1).

### 3.2. Interactions of THIQs Based on the Class of Antibiotics

The interactions were assessed using fractional inhibitory concentration indices (FICIs), isobolograms, and the proportion of each interaction against the MDR *S.* Typhi isolate. The combination of the five THIQs with the four antibiotics (20 combinations) resulted in at least one synergistic antibacterial effect per antibiotic class against a MDR *S.* Typhi clinical isolate.

Among all combinations tested, the overall percentage synergistic effects based on the total number of 7 MDR *S.* Typhi isolates ranged from 14.29 to 71.43%; additive effect ranged from 14.29 to 100%, antagonistic effect ranged from 71.43 to 85.71%, and no interaction effects ranged from 14.29 to 100% (Table 2).

Synergistic interactions were recorded for all four antibiotic classes. The highest level of synergistic interactions in terms of proportion of MDR bacterial isolates on which it was exerted is 71.4%, which was recorded for two combinations, i.e., chloramphenicol or sulphamethoxazole with compound **1**. This was followed by a moderate synergism for ampicillin with **1**, sulphamethoxazole-trimethoprim with **5**, and ciprofloxacin with **2** or **4**, which all recorded 57.1% (Table 2).

Additive interactions were recorded for all four antibiotic classes. The highest proportion of additive interactions recorded against all 7 isolates was 100% for ciprofloxacin with **1**, followed by chloramphenicol with compound **4** (71.4%). Moderate synergism was observed for ampicillin with **1**, chloramphenicol, or sulphamethoxazole-trimethoprim with **5**, which all recorded 42.8%. Also, all the THIQs showed additivity with sulphamethoxazole-trimethoprim.

**Figure 2:**
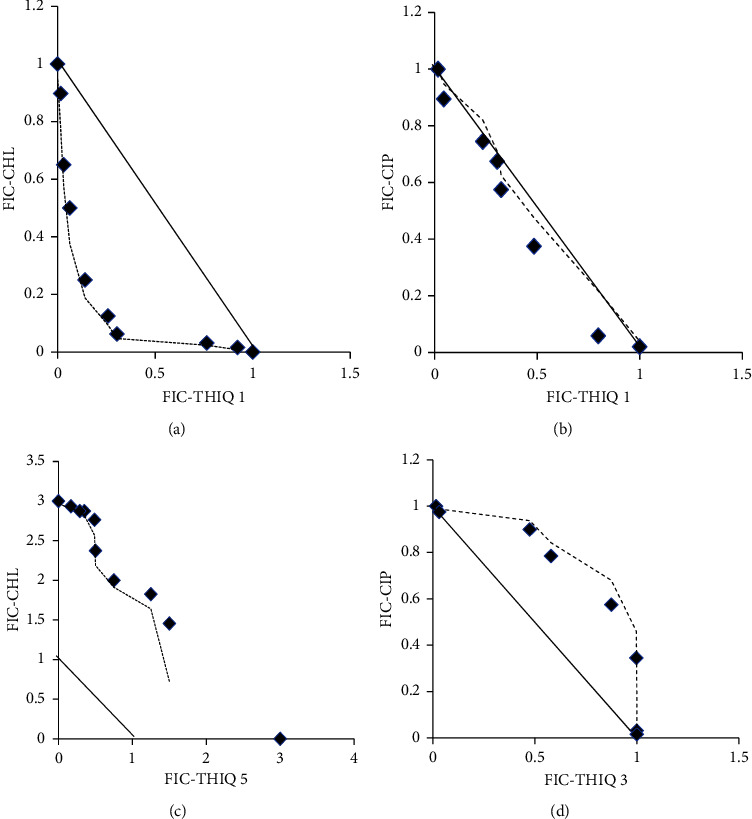
Isobolograms of combination effects of tetrahydroisoquinolines (THIQs), with treatment antibiotics against multidrug-resistant *S.* Typhi. (a) Synergistic effect (FICI = 0.078) of THIQ **1** and chloramphenicol (CHL), (b) additive effect (FICI = 0.625) of THIQ **1** and ciprofloxacin (CIP), (c) no interaction (indifference) (FICI = 1.25) of THIQ **5** and chloramphenicol, and (d) antagonistic effect (FICI = 4.25) of THIQ **3** and ciprofloxacin.

Antagonism was recorded in only one chemical class and for only two combinations (ciprofloxacin with **3** or **5**). Indifference (no interaction) was recorded in all antibiotic classes. These interactions are further illustrated in the isobolograms in Figure 2 for the most synergistic, additive, indifferent, and antagonistic THIQ-antibiotic combination against MDR *S.* Typhi.

Overall, in terms of the total of 20 THIQ-antibiotic combinations, seven (35%) and fourteen (70%) showed synergism and additivity, respectively (Table 2), with compound **1** being the most synergistic and additive in combination with three antibiotics (ampicillin, chloramphenicol, and sulphamethoxazole-trimethoprim).

## 4. Discussion

Various approaches are presently being used to counter increasing resistance in *Salmonella* including combination studies of active molecules. This study investigated the activity of combination treatment antibiotics with five tetrahydroisoquinolines (THIQs), which have shown moderate antibacterial activity against MDR *S.* Typhi strains. All five THIQs showed synergistic interaction with at least one class of treatment antibiotic. Overall, compound **1** was the most synergistic; it recorded synergistic activity with three antibiotic classes and the highest level of synergistic interactions (71.4%) against the total number of MDR *S.* Typhi isolates targeted. This is the first report of the synergistic activity of THIQs in combination with *Salmonella* treatment antibiotics.

The findings from this study confirm the multidrug-resistant nature of the *Salmonella* isolates as both THIQs and antibiotics recorded MICs in the same ranges as previously reported [8, 20] (Table 1). All THIQ-antibiotic combinations tested showed synergistic interactions against at least one isolate as seen in Table 2. THIQ **1** was the most synergistic in combination with chloramphenicol (Figure 2(a)) or sulphamethoxazole-trimethoprim and was not synergistic with ciprofloxacin. Furthermore, there was a massive reduction in the MIC of antibiotics, chloramphenicol, or sulphamethoxazole-trimethoprim, in combination with THIQ **1**, to the level recorded against sensitive *Salmonella* strains. This high reduction (64 fold) of the MIC of the treatment antibiotic further demonstrates the strong synergism in the combinations and indicates that THIQ **1** is a potential partner antibacterial which could be used in combination with some treatment antibiotics in the management of MDR *Salmonella* infections with resultant reduction in morbidity and mortality. However, further studies are required to establish this. The other four THIQs (**2**, **3**, **4**, and **5**) showed lower levels of synergism against the MDR *S.* Typhi strains in various combinations with ciprofloxacin and sulphamethoxazole-trimethoprim as shown in Table 2. The observed synergistic antibacterial activity could be due the action of the antibiotic and the THIQ at different targets in the bacterial cell; the differences in the level of synergism of THIQ **1** and the others could be attributed to their structures. As seen in Figure 1, when THIQ **1** with para-chloro substitution was combined with tested antibiotics, a synergistic effect with ampicillin, chloramphenicol, or sulphamethoxazole-trimethoprim was observed. The likely mechanism of action for the THIQ **1**-chloramphenicol combination could involve the inhibition of cell wall synthesis or some other essential process in the bacteria while chloramphenicol acts by its known mechanism of inhibition of protein synthesis [24]. In a previous work of seventeen THIQs [20] tested against MDR *Salmonella* strains including those used in this study, THIQ **1** showed the highest activity against MDR *Salmonella* strains; this activity was attributed to the electron-withdrawing property of the chloro substituent at the paraposition of the pendant phenyl [20]. This property may also account for the high synergism observed for this compound.

Additivity was also recorded in several combinations with four classes of antibiotics. High levels (70–100%) of additive interactions were recorded for THIQ **1** and **4** with three classes of antibiotics (Table 2, Figure 2(b)). Additive interactions suggest that the molecules in the combination may be sharing the same target sites, hence acting by the same mechanism in the bacterium, in which case both THIQ **1** and ciprofloxacin may be acting by inhibiting the bacterial DNA gyrase synthesis [24]. THIQ **1** also showed relatively lower additive interactions for all three antibiotic classes with which it was synergistic, further indicating that THIQ **1** may be acting at two different targets.

Antagonism was very low and was observed only for combinations of THIQs **3** (Figure 2(d)) and **5** with ciprofloxacin. THIQ **3** showed the highest indifference of 100% in combination with ampicillin or chloramphenicol. The likely mechanism of action here could be blocking of the site of action of the antibiotics by the THIQ in the cell wall for ampicillin and protein synthesis for chloramphenicol [24]. Antagonism suggests the THIQ prevents binding of antibiotic to its target while noninteraction suggests nonbinding of THIQ to the antibiotic target. The indifference and antagonism observed in THIQ **3** could be due to the presence of the di substitution (3,4-dichloro-) and THIQ **5** could be due to the presence of the methoxy (-OCH_3_) substituent on the pendant phenyl with lower electron-withdrawing at the paraposition resulting in decreased activity against MDR *S.* Typhi.

Overall, when all the interactions are considered, THIQ **1** was most synergistic and also showed relatively lower additive interaction, no antagonism, and no indifference; it showed the highest synergism with phenicols and antifolates. THIQ **4** was the most additive but showed indifference to a relatively lesser extent. However, **4** showed 100% additivity with ciprofloxacin; hence, it did not show synergism, antagonism, or indifference in this combination. The additivity observed in THIQ **4** combination with antibiotic may be due to the moderate electron withdrawing by the trifluoromethyl substituent at the paraposition as explained in [20].

Several studies of antibacterial compounds in combination with standard antibiotics have demonstrated improved activity against resistant strains of *S.* Typhi. However, most of these studies did not use the first-line treatment antibiotics. Miladi et al. [25] reported synergistic effect between thymol and nalidixic acid against nalidixic-resistant *S.* Typhimurium strains with the lowest MIC values ranging from 32 to 128 *μ*g/mL.

A study reported that the combination of erythromycin and epicatechin gallate against biofilm-forming MDR *S.* Typhimurium showed synergistic antibacterial effects with FIC indices of 0.5 [26]. Hence, it can be a potential treatment for *S.*Typhimurium-associated diarrhoea and its transmission from animals to humans.

Another study of plant-derived compounds (thymol and piperine) with three aminoglycosides (amikacin, kanamycin, and streptomycin) revealed strong synergistic effect against biofilm-forming resistant *Salmonella enterica* serovars (*S.* Typhi, *S.* Typhimurium, *S.* Enteritidis, and *S.* Choleraesuis). They also reported a 16-fold MIC reduction and potentiated the antibiofilm activity of aminoglycoside antibiotics [15].

Tetrandrine at subinhibitory concentrations in combination with colistin showed increased activity against most of the MCR-mediated colistin-resistant *Salmonella typhimurium*, with a FIC index (FICI) of 0.375–0.625 and the colistin MIC fold change ranged from 1 to ≥1024. They equally reported that the combination of tetrandrine and colistin demonstrated synergistic interactions in 81.8% (9/11) of the resistant *S.* Typhimurium isolates tested [27]. Hence, tetrandrine can serve as a potential colistin adjuvant against MCR-positive *Salmonella*.

In terms of strength, this study is the first to report synergistic activity of THIQs in combination with first-line antibiotics against MDR *Salmonella* strains. The practical value of this finding is that the highly synergistic combinations can potentially be used to treat MDR *Salmonella* infections. As limitation, the study design was based on previously published data and needed to assess reproducibility and validation of results. However, this limitation will be addressed in further exploitation of the findings of this work.

## 5. Conclusion

This study has revealed high synergistic and additive activities in combinations of tetrahydroisoquinoline with specific classes of treatment antibiotics against multidrug-resistant *Salmonella*. These active anti-*Salmonella* combinations are a potential alternative treatment for MDR *Salmonella* infections. These active combinations should be further tested *in vivo* to assess their efficacy and THIQs should also be studied in combination with other antibiotics classes.

## Figures and Tables

**Table 1:** Minimum inhibitory concentrations (MICs) and fractional inhibitory concentration indices (FICIs) showing effects of antibiotics in combination with THIQs.

Combination (THIQ/antibiotic)	MIC (*μ*g/mL)		MIC fold change (antibiotic)	FICc	FICa	FICI	Outcome
	MIC alone	MIC combined					
1/AMP	16/256	1/16	−16	0.0625	0.0625	0.125	*Synergism*
2/AMP	64/256	32/256	−1	0.5	1.0	1.5	Indifferent
3/AMP	256/256	64/256	−1	0.25	1.0	1.25	Indifferent
4/AMP	16/256	4/128	−2	0.25	0.5	0.75	Additive
5/AMP	256/256	256/256	−1	1.0	1.0	2.0	Indifferent
1/CHL	16/128	1/2	−64	0.0625	0.0156	0.078	Synergism
2/CHL	64/128	64/128	−1	1.0	1.0	2.0	Indifferent
3/CHL	256/128	128/128	−1	0.5	1.0	1.5	Indifferent
4/CHL	16/128	4/64	−2	0.25	0.5	0.75	Additive
5/CHL	256/128	64/128	−1	0.25	1.0	1.25	Indifferent
1/SXT	16/256	2/4	−64	0.125	0.0156	0.141	Synergism
2/SXT	64/256	64/256	−1	1.0	1.0	2.0	Indifferent
3/SXT	256/256	16/8	−32	0.0625	0.03125	0.094	Synergism
4/SXT	16/256	4/128	−2	0.25	0.5	0.75	Additive
5/SXT	256/256	64/64	−4	0.25	0.25	0.5	Synergism
1/CIP	16/16	2/8	−2	0.5	0.125	0.625	Additive
2/CIP	64/16	4/2	−16	0.0625	0.125	0.188	Synergism
3/CIP	256/16	128/64	+4	0.5	4.0	4.5	Antagonism
4/CIP	16/16	4/4	−4	0.25	0.25	0.5	Synergism
5/CIP	256/16	64/64	+4	0.25	4.0	4.25	Antagonism

THIQs: **1**, **2**, **3**, **4**, and **5**; antibiotics: AMP, ampicillin; CHL, chloramphenicol; SXT: sulphamethoxazole-trimethoprim; CIP: ciprofloxacin; FICa: fractional inhibitory concentration of antibiotic; FICc: fractional inhibitory concentration of compound; FICI: fractional inhibitory concentration index; −: MIC reduction; +: increase in MIC.

**Table 2:** Percentage drug interactions of tetrahydroisoquinolines (THIQs) and antibiotics against seven multidrug-resistant *Salmonella* isolates.

Combinations	No. of MDR *Salmonella* isolates per interaction (*n*) (%)			
	Synergism *n* (%)	Additivity *n* (%)	Antagonism *n* (%)	No effect *n* (%)
*Penicillins: ampicillin (AMP)*				
AMP-THIQ 1	4 (57.14)	3 (42.86)	0 (0)	0 (0)
AMP-THIQ 2	0 (0)	1 (14.29)	0 (0)	6 (85.71)
AMP-THIQ 3	0 (0)	0 (0)	0 (0)	7 (100)
AMP-THIQ 4	0 (0)	5 (71.43)	0 (0)	2 (28.57)
AMP-THIQ 5	0 (0)	1 (14.29)	0 (0)	6 (85.71)

*Phenicols: chloramphenicol (CHL)*				
CHL-THIQ 1	5 (71.43)	2 (28.57)	0 (0)	0 (0)
CHL-THIQ 2	0 (0)	2 (28.57)	0 (0)	5 (71.43)
CHL-THIQ 3	0 (0)	0 (0)	0 (0)	7 (100)
CHL-THIQ 4	0 (0)	5 (71.43)	0 (0)	2 (28.57)
CHL-THIQ 5	0 (0)	3 (42.86)	0 (0)	4 (57.14)

*Antifolates: sulphamethoxazole-trimethoprim (SXT)*				
SXT-THIQ 1	5 (71.43)	2 (28.57)	0 (0)	0 (0)
SXT-THIQ 2	0 (0)	2 (28.57)	0 (0)	5 (71.43)
SXT-THIQ 3	1 (14.29)	1 (14.29)	0 (0)	5 (71.43)
SXT-THIQ 4	0 (0)	5 (71.43)	0 (0)	2 (28.57)
SXT-THIQ 5	4 (57.14)	3 (42.86)	0 (0)	0 (0)

*Fluoroquinolones: ciprofloxacin (CIP)*				
CIP-THIQ 1	0 (0)	7 (100)	0 (0)	0 (0)
CIP-THIQ 2	4 (57.14)	0 (0)	0 (0)	3 (42.86)
CIP-THIQ 3	0 (0)	0 (0)	5 (71.43)	2 (28.57)
CIP-THIQ 4	4 (57.14)	0 (0)	0 (0)	3 (42.86)
CIP-THIQ 5	0 (0)	0 (0)	6 (85.71)	1 (14.29)

^*∗*^Total assays (140)	27 (17)	42 (30)	11 (7.8)	60 (42)

Total combinations (20)	7 (35)	14 (70)	2 (10)	15 (75)

*n* = number of MDR *Salmonella* isolates which showed a given interaction; ^*∗*^Distribution of interaction type based on the total number of THIQ-antibiotic combination assays against 7 MDR *Salmonella* isolates.

## Data Availability

The data used to support the findings of this study are available from the corresponding author upon request.
